# A Cross-Sectional Survey on the Impact of Irrelevant Speech Noise on Annoyance, Mental Health and Well-being, Performance and Occupants’ Behavior in Shared and Open-Plan Offices

**DOI:** 10.3390/ijerph16020280

**Published:** 2019-01-19

**Authors:** Sonja Di Blasio, Louena Shtrepi, Giuseppina Emma Puglisi, Arianna Astolfi

**Affiliations:** Department of Energy, Politecnico di Torino, Corso Duca degli Abruzzi 24, 10129 Torino, Italy; louena.shtrepi@polito.it (L.S.); giuseppina.puglisi@polito.it (G.E.P.); arianna.astolfi@polito.it (A.A.)

**Keywords:** irrelevant speech noise, noise annoyance, productivity, mental health, well-being, cross-sectional survey, open-plan offices, shared offices, occupants’ behavior

## Abstract

This cross-sectional survey has compared subjective outcomes obtained from workers in shared (2–5 occupants) and open-plan (+5 occupants) offices, related to irrelevant speech, which is the noise that is generated from conversations between colleagues, telephone calls and laughter. Answers from 1078 subjects (55% in shared offices and 45% in open-plan offices) have shown that irrelevant speech increases noise annoyance, decreases work performance, and increases symptoms related to mental health and well-being more in open-plan than in shared offices. Workers often use headphones with music to contrast irrelevant speech in open-plan offices, while they take a break, change their working space, close the door or work from home in shared offices. Being female, when there are more than 20 occupants, and working in southern cities without acoustic treatments in the office, make it more likely for the occupants to be annoyed by irrelevant speech noise in open-plan offices. While, working in southern cities and with acoustic treatments in the office makes it more likely that noise annoyance will be reported in shared offices. Finally, more than 70% of the interviewed in open-plan offices were willing to reduce their voice volumes when advised by a noise monitoring system with a lighting feedback.

## 1. Introduction

Irrelevant speech noise (ISN) is the noise that is generated from conversations between colleagues, telephone calls and laughter [1,2]. Office workers have mentioned that irrelevant speech is the most disturbing source of noise [1,3,4,5,6] in open-plan offices, due to the overall noise level and intelligible conversations [7,8,9]. A large number of cross-sectional surveys have been carried out on the theme of noise and comfort in open plan offices, but none has focused on ISN, although it has been reported to be the main source of annoyance and dissatisfaction. Moreover, no investigation on noise perception and its effects in offices with different sizes has been performed yet. In fact, the investigations so far performed have been aimed at considering the differences between open-plan and cellular offices, the latter with from one to two people, but shared offices that seat from two to five people, which can be alternatives to very noisy open-plan offices, have not been considered.

Given the relevance of the consequences of noise in offices with more than two occupants, several studies have investigated solutions for its mitigation, one of which is the room acoustic treatment.

The background on annoyance, loss of performance, mental health and well-being, due to noise in different types of offices is summarized hereafter, together with information on the effectiveness of acoustic interventions in rooms.

### 1.1. Background on Annoyance, Loss of Performance and Mental Health, and Well-Being from Noise in Offices

Several studies have dealt with the effects of noise annoyance in offices. Noise annoyance has been defined as a multi-faceted concept that includes behavioral noise effects, such as disturbance, and interferes with intended activities and evaluative aspects, such as nuisance, unpleasantness and getting on one’s nerves [10]. In their semantic study, Guski et al. [10] demonstrated that the concept of noise annoyance was closely associated with disturbance and nuisance concepts. Therefore, the term “annoyance” has been used in the present study to indicate one effect of ISN.

Based on subjective assessments, a self-estimated loss of performance, due to ISN, was found in open-plan offices [1,5]. This outcome was then confirmed from the results of laboratory studies where subjective perception was considered and tests over several cognitive tasks were performed [8,11,12,13].

A considerable amount of literature has been published on the relationships between the presence of office noise, mental health and well-being. According to the WHO [14], mental health is a state of well-being in which the individual realizes his or her own abilities, can cope with the normal stresses of life, can work productively and fruitfully, and is able to make a contribution to his or her community. Mental wellbeing has instead been defined simply as feeling good and functioning well [15]. The consequences of mental health problems in the workplace can be depression, stress, burnout, but also headaches, ulcers, high blood pressure, reduction in productivity and output, loss of motivation and commitment, tension and conflicts between colleagues, etc. [16]. Several studies have investigated the typical symptoms and feelings associated with mental health and well-being in open-plan offices. Numerous symptoms, such as fatigue and headaches [1,17], difficulties in concentration [1,4,17], physiological stress [18], loss of motivation and tiredness [8] and increased cognitive workload [19], have been perceived in open-plan offices. Jahncke et al. [8] showed that the self-rating of the tiredness and motivation of subjects decreases for high noise levels compared to low noise levels. Pejtersen et al. [17] specified that fatigue, headaches and difficulties in concentration are related to the office size. De Croon et al. [19] highlighted that the cognitive workload increases as a result of the openness of a workplace and the distance between workstations. Moreover, Pejtersen et al. [17] and Denielsson [20] reported an increased sickness absence in open-plan offices.

### 1.2. Surveys on the Effects of Noise in Different Types of Office

A large number of studies have conducted subjective surveys on the effect of noise in open-plan offices [2,6,11,19], while others have performed comparisons between private and open-plan offices [1,5], but only a few have dealt with comparisons between different sized offices [17,20,21]. Moreover, about half of them carried out subjective surveys rather than objective ones, such acoustical measurements and cognitive tasks. Indeed, as affirmed by Frontczak et al. [22], occupants are the best source of information in terms of needs and comfort requirements.

In a cross-sectional survey on over 2300 workers, Pejtersen et al. [17] showed that noise was significantly related to the size of an office. They pointed out that occupants’ complaints about noise increased tenfold in open-plan offices, compared to cellular offices. Denielsson [20] also found that noise disturbance was higher in large open-plan offices than in cellular ones. In a questionnaire survey carried out in office buildings, Sakellaris et al. [23] found that noise inside the building was closely related to the occupants’ overall comfort, which in turn was affected by gender and age, and by building features, such as office size and building location.

### 1.3. The Influence of Room Acoustics on the Reduction and Perception of Noise in Offices

Some studies have focused on how to apply room acoustic solutions, such as sound absorption materials, screens between workstations [9,11,24,25] and sound masking systems [9,11,26,27] in offices in order to improve the acoustic conditions and reduce noise. As Hongisto et al. [22] declared, very little is currently known about the effects of room acoustics on the reduction of ISN, while Haapakangas et al. [11] found that disturbance, due to intelligible background speech, can be reduced by an optimal and accurate acoustic design of the office when the speaker and listener are at least four-to-six meters away from each other.

The present study is the first to have carried out a cross-sectional survey in order to specifically explore the impact of ISN on annoyance, performance, mental health and well-being, and occupants’ behavior through a self-assessment questionnaire in two types of office, that is, shared and open-plan offices. Furthermore, it explores the willingness of office occupants to reduce ISN through their active involvement, which means lowering their voice volume when advised to do so by a noise monitoring system with lighting feedback, for example, Speech and Sound SEMaphore (SEM) [28] or SoundEar [29]. This research question arises from the studies of Hongisto et al. [25], who affirmed that one of the ways to reduce the disturbance of speech is to lower the voice effort. In addition, Bradley [30] considered that office etiquette was a successful way of encouraging the use of low voice levels in open-plan offices, and Schlittmeier and Liebl [9] affirmed that social conventions, such as defined silent times and phone times, can help to limit noise levels resulting from speech in open-plan offices.

Three research aims have been addressed in this work: (i) to evaluate the effects of ISN on annoyance, performance, mental health and well-being, and occupants’ behavior in shared and open-plan offices; (ii) to investigate the relationships between perceived noise annoyance and personal characteristics (age, gender and professional sector) and office characteristics (city, number of people in the office and room acoustic design); (iii) to evaluate the attitude of workers toward the use of a noise monitoring system with lighting feedback to encourage new adaptive behavior in open-plan offices, such as controlling voice level.

## 2. Materials and Methods

### 2.1. Subjects and Offices

Nineteen companies, including eleven small and eight large companies, five research centers and one university in Italy were involved in the cross-sectional survey. The selected offices differed in terms of city, type of activity carried out by the workers, office layout and room acoustic design. The location of the cities was considered, since there is evidence of the multiple socio-economic-cultural variables that characterize the north-south differential [31]. A total of 1180 employees were recruited, through an online questionnaire, from September to November 2017. A response rate of 17% was achieved, which is in line with the response rate of online questionnaires [32]. Since the survey was aimed at investigating the effects of ISN inside offices, the responses of 102 employees who worked in private offices were excluded because the source of noise was mainly related to speech sound coming from outside the office and useful speech from colleagues visiting the office. Consequently, 1078 out of the 1180 subjects were considered for the analysis based on completed questionnaire, which were only registered in the database. The total sample (N = 1078) was divided into two samples according to the classification of the offices based on layout. Sample (S) included answers from subjects working in shared offices (from two to five workers), while sample (O) referred to open-plan offices (more than five people) [25]. Therefore, the corresponding percentages were about 55% (597 subjects) in the shared offices and about 45% (481 subjects) in the open-plan offices. As far as the total sample is concerned, 55% of the subjects worked in university, 3% in research centers, 41% in companies and 1% of the subjects were freelance workers; 28% of the workers came from engineering sectors, 21% from technical sectors and 27% from the administration sector. Background information, related to the city where the office was located, age, gender, professional sectors and number of workers in the office, is shown in Table 1. The respondents were 58% male and 42% female. A total of 78% of the subjects worked in Turin. The total sample was mainly distributed into three age ranges: 26–35 (33%), 36–50 (26%) and 51–65 years old (36%). The percentage of subjects that worked in shared, medium and large open-plan offices was about 55%, 43% and 1%, respectively.

### 2.2. Questionnaire

The questionnaire was prepared through Google Forms [33] and administrated through an online link distributed by email. It was designed according to the ethical code of the authors’ university. The head of the human resources of each company approved the questionnaire. An accompanying letter was added to the email to invite workers to voluntarily participate in the survey. The subjects were also informed, in the letter, about the confidential treatment of their personal data and about the anonymity of the answers. 

The online questionnaire included 17 questions, and it was available in Italian and English. Less than 5 min was needed to fill it in, a time that was chosen in order to avoid overtaxing and high dropout rates because of boredom.

The questionnaire was composed of three sections: (1) an explanation of the aim of the survey and response time (2) background questions (3) subjective opinions. The aim of the study was explained in the accompanying letter of the first section and the definition of ISN was provided, i.e., “the noise generated from conversations between colleagues, telephone calls and laughter” [1,2]. The term “chatting noise” was used in the questions instead of “Irrelevant Speech Noise” as it is a more common term and it is easier to understand by lay respondents. The seven background questions were submitted in order to collect general information about the gender, age, nationality, company and professional sector of the respondents.

The 10 questions in the third section are shown in Table 2. The following topics were investigated: annoyance (Q1), mental health and well-being (Q2 and Q6), productivity (Q3–Q5), occupants’ behavior (Q7 and Q10) and the presence of acoustic treatments (Q8 and Q9). Several feelings and symptoms related to mental health and well-being were presented to the subjects in Q2 [14,16,34]. This list of feelings and symptoms can be divided as follows: (1) mental illness, such as stress, (2) loss of concentration, (3) emotional and social feelings, such as feeling displeased, loss of motivation, anger, negative feelings towards colleagues, and (4) physical symptoms, such as tiredness, overstrain and headaches. In addition, since mental health and well-being are closely related to interpersonal relationships [16], this aspect was investigated in Q6.

Occupants’ behavior adopted to cope with ISN was assessed in Q7 and Q10. The personal strategies used to reduce annoyance resulting from people chatting were investigated in Q7 considering the following items: (1) use of technological tools, such as headphones with music and noise cancelling headphones, and (2) use of adaptive behavior [35,36], such as taking a break, changing working space, changing work task, working from home and closing the office door, and (3) asking colleagues to reduce their voice levels. The willingness of workers to be actively involved in ISN reduction, by lowering their voice volume when advised by a light-system device, was investigated in Q10 as a further feature of the occupants’ behavior.

The content of the questionnaire was explicitly defined according to the purpose of the study. The wording of the questions, as well as the Likert scale ranking and the list of alternatives was drawn up on the basis of previous studies. Q1 and Q3–Q6 were based on questions presented in [25] and [26], respectively. The single choice questions, Q2 and Q7, were included according to [37,38], with the aim of investigating the main feeling or symptom and the main personal strategy adopted with regard to ISN, respectively. The lists of alternatives were defined according to [1,16,17] for Q2 and to [1] for Q7. Q8, Q9 and Q10 are new, compared to previous studies, and the list of alternatives in Q9 were designed considering the acoustic treatments generally used in offices. The options in Q2, Q7 and Q9 were randomized to change the order in which they were presented to the subjects.

### 2.3. Statistical Analysis

A statistical analysis was carried out with SPSS (IBM Statistics20, IBM, Armonk, NY, USA). According to Siegel and Castellan [39], non-parametric methods should be used to analyze data measured with ordinal and nominal scales, as in the case of this study. The significance of the differences between shared and open-plan offices, related to several factors such as noise annoyance, mental health and well-being, and work productivity, was assessed with the Mann-Whitney U Test (MWU), a test that is used for two groups of independent observations. This test was also applied for both types of office to investigate how noise annoyance varied according to gender, location of the city, and the presence or absence of acoustic treatments. The Kruskal-Wallis (KW) test, which is an extension of the MWU test for more than two groups, was applied in order to investigate how noise annoyance is related to different age ranges, professional sectors and number of people in an office. Subsequently, when a significant difference was found between groups, the Mann-Whitney U Test was applied between paired groups.

A logistic regression analysis was then performed in order to investigate the personal and office characteristics that affect noise annoyance, such as gender, age, city, professional sector, acoustic treatment and number of people in the office, in both shared and open-plan offices. Significant covariates were identified in the models on the basis of the “forward” variable selection procedure [40]. The “noise annoyance” response variable was dichotomized into “no annoyance” (1 = not at all; 2 = slightly) and “annoyance” (3 = fairly; 4 = highly; 5 = extremely).

Finally, the significance of the differences between shared and open-plan offices was detected by the z-test for proportions [41] when a nominal scale was used to gather data, as for Q2 and Q7.

## 3. Results

### 3.1. Effects of ISN on Annoyance, Productivity, Mental Health and Well-Being

Table 3 shows lower mean and mode values of Q1 to Q6 in shared offices compared to open-plan ones and significant differences between the two office types, according to the MWU Test. The workers in shared offices perceive ISN as less annoying than the workers in open-plan offices, and ISN compromises work performance and interpersonal relationships between colleagues less in shared offices than in open-plan ones. Significant positive correlations, with Spearman coefficients ≥0.5 and *p*-values < 0.01, were also found between the noise annoyance scores for Q1 and the scores for Q3 to Q6 in both shared and open-plan offices.

### 3.2. Effects of ISN on Mental Health and Well-being, and Occupants’ Behavior

**Figure 1:**
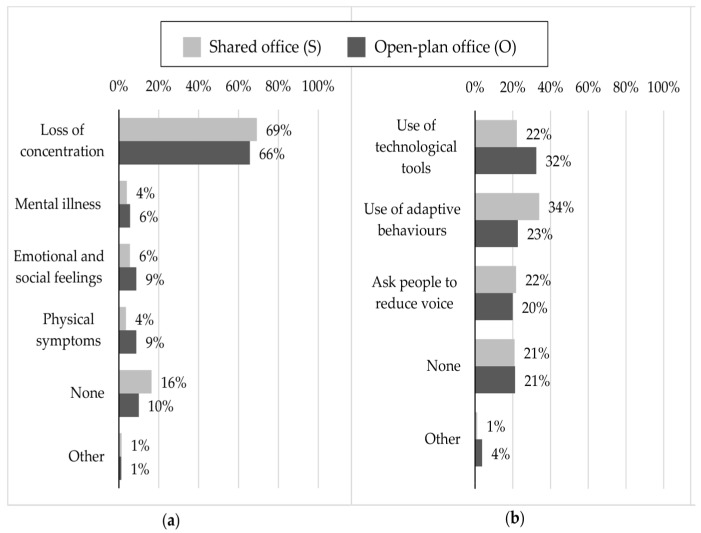
Percentages related to the effects of ISN on mental health and well-being, and occupants’ behavior in shared (S) and open-plan (O) offices: (**a**) Subjective ratings on feelings and symptoms attributed by occupants to ISN; (**b**) Subjective ratings on personal strategies used by occupants to cope with ISN.

Figure 1a shows the percentages of the feelings and symptoms indicated by the employees as a consequence of ISN, in shared and open-plan offices. It emerged that 69% and 66% of the employees declared that a loss of concentration is the main feeling consequence of ISN in shared and open-plan offices, respectively, while 4% and 6% of the workers self-estimated mental illness, such as stress, as the main feeling consequence of ISN in office types S and O, respectively. Emotional and social feelings, such as feeling displeased, less motivated, angry and negative feeling toward colleagues, were found to be the main consequence of ISN for 6% and 9% of the workers in office types S and O, respectively. Similarly, 4% and 9% of the workers related ISN with physical symptoms, such as tiredness, overstrain and headaches, in office types S and O, respectively. Significant differences were found between shared and open-plan offices, according to the z-test for proportions, for emotional and social feelings and physical symptoms (z equal to 2.06 and 3.63, respectively; *p*-value = 0.05).

Figure 1b shows the percentages related to different strategies adopted by the workers in order to cope with ISN in shared and open-plan offices, respectively. The use of technological tools, such as headphones with music and noise cancelling headphones, was the main solution for 22% and 32% of the workers in the S and O office types, respectively. Taking a break, changing working space or work task, working from home and closing the office door were the main strategies, in the form of adaptive behavior, adopted by 34% and 23% of the workers in S and O, respectively. Furthermore, 22% and 20% of the employees preferred asking colleagues to reduce their voice levels in office types S and O, respectively. Significant differences, with lower *p*-values than 0.05, were found between shared and open-plan offices, according to the z-test for proportions, but only related to the technological tools and adaptive behaviors (z equal to 3.81 and –4.08, respectively; *p*-value = 0.05) used by workers to cope with ISN.

**Figure 2:**
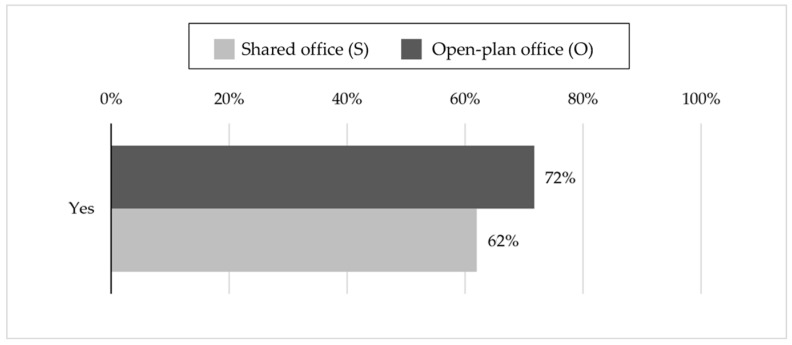
Percentages related to the willingness of the occupants to be influenced by a noise monitoring system with lighting feedback that encourages behavioral changes, such as the decrease of the voice volumes in order to reduce ISN.

A total of 62% of the workers in shared offices and 72% in open-plan ones declared they were willing to reduce their voice levels when advised by a light-system device, which monitors the ISN level in shared and open-plan offices, as shown in Figure 2. Such a difference between offices was found to be statistically significant, according to the z-test for proportions (z equal to 3.37; *p*-value = 0.05).

### 3.3. Noise Annoyance, and Personal and Office Characteristics

#### 3.3.1. Gender, Age Range, Professional Sectors and City

Table 4 shows the mean and mode values of the noise annoyance scores divided according to gender, age range, professional sector and city location, for both shared and open-plan offices, together with the significance of the differences between groups, according to the MWU or KW Test.

The analyses yielded no significant difference between genders, according to the MWU Test, in the shared offices. This trend is also supported by the fact that the same mode value emerged for both genders. Conversely, a significant difference was found in open-plan offices, when observing the highest mean and mode values, with women being more annoyed by ISN than men.

Significant differences were observed between the three age ranges, that is, 18–25, 26–35 and 51–65+, although only in open-plan offices, according to the KW Test. A significant difference was observed between the first range, 18–35 years, and the last range, 51–65+ years, according to the MWU Test, with older subjects being more annoyed, i.e., with higher mean values.

No significant differences between professional sectors emerged, according to the KW Test, between the shared offices and the open-plan offices. However, the highest and lowest mean values were found for the subjects that work in the sales and public affairs sector (SPA) and in the engineering and teaching sector (EN-TE), in both types of office. However, the low number of subjects involved in the public affairs sector affected the statistical results of this sector, and further investigations are required.

The cities were divided in northern and southern cities based on their location. The analyses provided significant differences between the two locations of the cities in both shared and open-plan offices, with higher mean values for the subjects who work in southern cities than those who work in northern cities.

#### 3.3.2. Number of People in the Office 

Significant differences between shared (S), medium open-plan (MO) and large open-plan (LO) offices were obtained from the KW Test, as shown in Table 5, and significant differences between each paired office type were also found for the MWU Test. The mean values generally show that workers are more annoyed by ISN as the number of people in offices increases.

#### 3.3.3. Presence of Acoustic Treatments

**Figure 3:**
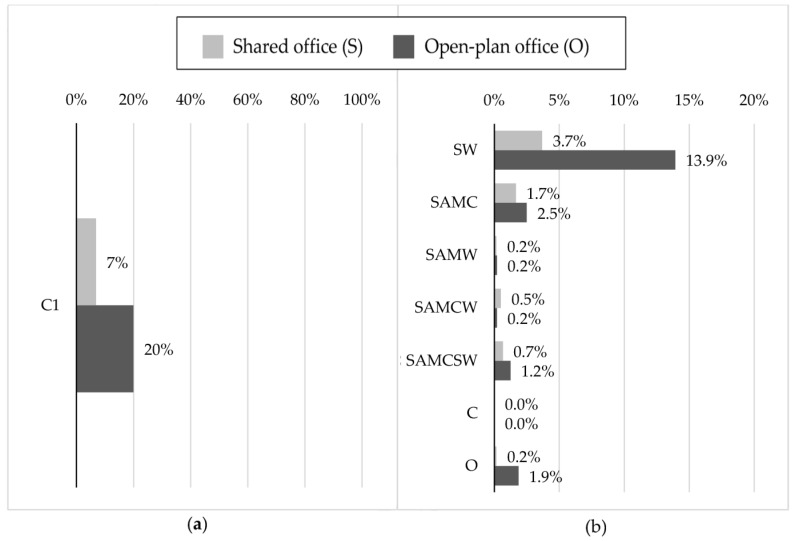
(**a**) Percentages of acoustic treatments self-estimated by the subjects for the shared and open-plan offices: (**a**) Subjective ratings on the presence (C1) of acoustic treatments; (**b**) Subjective ratings on the acoustic treatment types. The following abbreviations were adopted: SW for “screens between workstation”, SAMC for “sound absorption materials on ceiling”, SAMW for “sound absorption materials on walls”, SAMCW for “sound absorption materials on ceiling and walls”, C for “carpets”, SAMCSW for “sound absorption materials on ceiling with screens between workstations” and O for “other”.

The subjects were asked to indicate whether any acoustic treatments were present in their office, after a visual inspection. As shown in Figure 3a, according to the respondents, 7% and 20% of the shared and open-plan offices were acoustically treated. As can be observed in Figure 3b, setting screens between workstations (SW) is the most commonly adopted acoustic treatment, and this is followed by the application of sound absorption materials on the ceiling (SAMC).

As can be observed in Table 6, significant difference emerged between the noise annoyance scores for the workers who self-estimated the presence (C1) or absence (C2) of acoustic treatment in open-plan offices, according to the MWU Test. Workers were found to be significantly more annoyed by ISN when open-plan offices were not acoustically treated, as shown by the higher mean and mode values.

#### 3.3.4. Personal and Office Characteristics that Affect Noise Annoyance

Table 7 shows the Odds Ratio (OR) of the effects of a number of covariates that significantly affect noise annoyance, according to the logistic regression analysis, for both types of office. The included variables are categorial, and the reference categories are the ones with the lowest mean noise annoyance score shown in Table 4, Table 5 and Table 6.

The regression model identified the location of the city, and the acoustic treatment as the significant covariates that affect noise annoyance in shared offices. Office workers in southern cities and workers with acoustic treatments in the office are about two times more likely to report annoyance from ISN than subjects who work in northern cities and in offices without acoustic treatments.

In addition to the location and the acoustic treatment, the regression model related to open-plan offices identified the gender and the number of people in the office as further significant covariates. Being female and working in southern cities, without acoustic treatment in the office, makes it about two times more likely to be annoyed by ISN than being male, working in northern cities and with acoustic treatment in the office. Furthermore, when there are more than 20 people in the office, it is about nine times more probable that the workers will be annoyed by ISN than when there are from 6 to 20 occupants.

The outcome related to the acoustic treatment condition in the office was found to be the opposite for the two office types, in agreement with the mean values shown in Table 6. The occupants of shared offices are less annoyed by ISN when the office is without any acoustic treatment, while the occupants of open-plan offices are less annoyed when the office has acoustical treatment.

## 4. Discussion

Using data from the survey, which was administrated in eleven small and eight large companies, five research centers and one university in Italy, the present study has investigated the effects of irrelevant speech noise (ISN) in shared (two to five employees), and open-plan offices (more than five employees).

Although many surveys have been conducted on noise annoyance and acoustic comfort in open-plan offices, this study is the first that has specifically explored the impact of ISN on annoyance, performance, mental health and well-being and occupants’ behavior. Moreover, this is the first study that has compared subjective assessments of shared offices with those of open-plan offices. The associations between perceived noise annoyance and personal characteristics (age, gender and professional sector) and office characteristics (city, number of people and room acoustics design) have also been evaluated. In addition, the study has investigated the willingness of office occupants to reduce ISN by lowering their voice levels when a noise monitoring system with lighting feedback advises them that high noise levels have been reached.

### 4.1. Effects of ISN on Noise Annoyance, Productivity, and Mental Helath and Well-Being

This study highlights that ISN generated by conversations between colleagues, telephone calls and laughter was more annoying in open-plan offices than in shared ones, and workers showed an increased annoyance with ISN according to the office size, i.e., in shared, medium and large open-plan offices. This result is in line with Danielsson [20], who found that noise due to conversation, equipment and other office noise sources was more annoying in open-plan offices than in smaller ones.

The perceived decrease in work productivity as a result of ISN was higher in open-plan offices than in shared offices. Workers were often interrupted by ISN and consequently they were not able to maximize their performance in larger offices. This comparison between shared and open-plan offices has not been investigated in the previous studies. However, it is coherent with the results of Kaarlela-Tuomaala et al. [1], who showed that the self-estimated waste of working time, due to noise, doubled when workers moved from private to open-plan offices. This result is important, in terms of office layout, since shared offices can be considered as an alternative to open-plan offices when the work productivity has to be increased.

About 70% of the workers in both office types declared difficulties in concentration as the self-estimated main feeling caused by ISN, with no significant difference between the types of offices. On the other hand, the feelings and symtoms related to mental health and well-being, such as emotional and social feelings (feeling displeased, less motivated, angry and feeling negative toward colleagues) and physical symptoms (tiredness, overstrain and headaches), were in general mentioned less frequently by the workers as the main consequences of ISN, but a significant increase was found for open-plan offices compared to shared offices. The pattern of results is in line with previous studies that demonstrated an association between the noise of different types of office and difficulties in concentration [1,4,17], and other feelings and symptoms related to mental health and well-being, such as tiredness and motivation [8], fatigue and headaches [1,17]. Moreover, the results are in agreement with those of Pejtersen et al. [17], who observed a significant increase in the prevalence of physical symptoms, such as fatigue and headaches, according to the size of the office.

In addition, mental health and well-being, related to interpersonal relationships between colleagues, was found to be more affected by ISN in open-plan offices than in shared ones. This finding is coherent with that of Brennan et al. [21], who underlined a worsening of satisfaction in co-worker relationships when they moved from private to shared offices.

### 4.2. Effects of ISN on Occupants’ Behavior

One finding of the present work is that there was a significant difference between shared and open-plan offices, in terms of strategies adopted to cope with ISN, thus highlighting important implications for the design and management of offices. The use of technological tools, such as headphones with music, was the main solution used by workers to contrast ISN in open-plan offices, while adaptive behaviors, such as taking a break, changing working space or work task, working from home and closing the office door were the main strategies used in shared offices. Similar outcomes were also highlighted by Kaarlela-Tuomaala et al. [1], who found that workers adopted more coping strategies in open-plan offices than in private offices.

An important finding of this study is that about 70% of the workers in the open-plan offices and about 60% in the shared offices were willing to reduce ISN through their active involvement, if a noise monitoring system, with lighting feedback, advised them to reduce their voice volumes. The percentage was significantly higher for open-plan offices than shared offices, perhaps as a result of the higher perceived noise annoyance. According to these outcomes, it is possible to state that since an accurate room acoustic design is not enough to reduce the distraction and annoyance generated by nearby speech sounds [11,25], the use of a noise monitoring system, with lighting feedback, could be an effective complementary method. In this way, a positive behavior of the occupants would be promoted, such as lowering their voice levels or changing the room where they chat. In addition to the already experimented passive measures introduced to reduce ISN, such as the room acoustic design [9,11,24,25] or sound masking [9,26,27], this system may be able to reinforce and promote office etiquette and a behavioral code.

### 4.3. Effects Of Personal and Office Characteristics on Noise Annoyance

Women were found to be significantly more annoyed than men in open-plan offices, while no differences were found for shared offices. This outcome is in line with those of Kaarlela et al. [1] and Danielsson et al. [42], who found that women were more disturbed by noise than men in open-plan offices. Significant differences between noise annoyance scores for three age ranges were also obtained, although only in open-plan offices. A significant difference between the first range of 18–35 years and the last range of 51–65+ years was found, with the older subjects being more annoyed. In line with this finding, Pierrette et al. [6] found a significant positive correlation between age and perceived annoyance due to ISN, while Sakellaries et al. [23] documented that noise was an important factor for the oldest workers in open-plan offices.

No significant differences between professional sectors was obtained for the shared and open-plan offices related to noise annoyance due to ISN, even though the highest and lowest mean values of noise annoyance were found for subjects that worked in the sales and public affairs sector (SPA) and in the engineering and teaching sector (EN-TE). Since it is possible to hypothesize that engineers tend to carry out counting tasks, this result is in line with a previous finding by Logie [43], who showed that people who did mathematical tasks suffered a great deal from speech noise.

Significant differences between location of the cities were obtained for both the shared and open-plan offices. The results showed that subjects working in southern cities were more annoyed than subjects working in northern ones.

According to the workers’ assessments, a small percentage of offices were acoustically treated in the study sites. The respondents identified screens between workstations as the most frequently used acoustic treatment in open-plan offices. Nevertheless, workers were found to be significantly more annoyed by ISN when open-plan offices were not acoustically treated, while the opposite emerged for shared offices. Seddigh et al. [24] showed that improved room acoustics was associated with lower perceived disturbances and cognitive stress in open-plan offices; however, in order to prove this aspect in the present study, it would be necessary to conduct objective measurements of noise levels and obtain further information about the introduced acoustic treatments.

When all the above-mentioned characteristics were considered together in the evaluation of the most important factors that affect noise annoyance in shared and open-plan offices, some of them resulted to be more significant. Office location and the room acoustic design were the most significant factors that affected the perception of noise annoyance in both office types, while gender and the number of people in the office also significantly affected noise annoyance in open-plan offices. It is important to underline that the acoustic treatment of rooms affects noise annoyance in open-plan and shared offices in different ways. Noise annoyance was reduced as the result of an acoustical treatment in the former and without any acoustical treatment in the latter. The ISN level is generally lower in shared offices than in open-plan offices, due to the presence of fewer people, but speech privacy can also be reduced. Speech privacy increases when reverberation increases [44], and for this reason the occupants could have identified a more reverberant environment as less annoying, because it resulted in a less intelligible speech.

In order to investigate these aspects, future research will deal with the same type of investigation inside the same offices types, but with the addition of acoustic measurements in both acoustically treated and untreated offices, and with or without the presence of a light system that advises people to reduce their voice volumes.

### 4.4. Limitations

The present study suffers from some limitations. Caution is needed when comparing the results of this work with the findings of previous studies, since the self-assessment questionnaire has not been suitably validated according to e.g., [37,38,45]. Nevertheless, an exploration stage was introduced according to [38], where people with special expertise in acoustics and architecture and people from the target population were involved in order to identify any ambiguities in the questions and to determine the list of possible responses for the proposed alternatives. Moreover, some questions were taken from distinguished literature that used validated questionnaires. Another limitation is that other factors that can affect noise annoyance, such as noise sensitivity [46], personal attitudes and psychosocial factors [10], were not taken into consideration. Another weakness is related to the cross-sectional study method, which does not allow the causality of the identified association between ISN and several of the investigated factors to be established [1]. Furthermore, certain limitations can be highlighted as a result of resorting to an online survey [47]. The nonresponse bias, in particular, cannot be investigated online, since the identity of non-respondents is generally unknown [48]. There is also a self-selection bias, i.e., subjects that were more annoyed by ISN in their offices may have been more likely to complete the questionnaire than those who were not so annoyed. This bias could be particularly marked duo to the low response rate. Moreover, the multiple responses of subjects cannot be excluded.

## 5. Conclusions

This cross-sectional study has compared the subjective outcomes of shared offices (2–5 workers) and open-plan offices (+5 workers) related to “irrelevant speech noise”, which is the noise generated from conversations between colleagues, telephone calls and laughter. Answers from an online questionnaire were collected in nineteen companies, five research centres and one university in Italy, from 1078 subjects, of which 55% worked in shared offices and 45% in open-plan offices.

Irrelevant speech noise was found to be more annoying in open-plan offices and it compromise performance, mental health and well-being more than in shared offices. In open-plan offices, being female, and working in the southern cities without any acoustic treatment in the office, made it more likely for the respondents to be annoyed by irrelevant speech noise than being male and working in the northern cities with acoustic treatment in the office. Furthermore, having more than 20 occupants in an office made being annoyed more probable than having from 6 to 20 occupants. Moreover, working in the southern cities and with acoustic treatment in the office made it more likely that noise annoyance will be reported in shared offices.

Headphones with music was found to be the main solution adopted by workers to contrast irrelevant speech noise in open-plan offices, while adaptive behaviors, such as taking a break, changing working space or work task, working from home and closing the office door were the main strategies used in shared offices. A high percentage of workers stated they were willing to reduce irrelevant speech noise if a noise monitoring system with lighting feedback advised them to reduce their voice volumes.

## Figures and Tables

**Table 1:** Main characteristics of the total sample (N = 1078) subdivided into shared and open-plan offices. The percentages of the two samples are indicated in square brackets.

Background Information		Shared Offices	Open-Plan Offices
**Gender**	Female	269 (45)	188 (39)
	Male	328 (55)	293 (61)
**City**	Milan	11 (2)	28 (6)
	Turin	464 (78)	378 (79)
	Cuneo	5 (1)	10 (2)
	Rome	27 (5)	31 (6)
	Naples	88 (15)	34 (7)
	Other	2 (0)	0 (0)
**Age range**	18–25	23 (4)	26 (5)
	26–35	170 (28)	182 (38)
	36–50	187 (31)	98 (20)
	51–65	212 (36)	175 (36)
	65+	5 (1)	0 (0)
**Professional sector**	Technical	118 (20)	104 (22)
	Engineering	177 (30)	124 (26)
	Management	42 (7)	37 (8)
	Administration	152 (25)	139 (29)
	Creative, design and architecture	46 (8)	30 (6)
	Sales and public affairs	9 (2)	20 (4)
	Teaching	4 (1)	2 (0)
	Other	49 (8)	25 (5)
**Number of people in the offices**	From 2 to 5 (shared)	597 (55)	-
	From 6 to 20 (medium open-plan)	-	467 (43)
	From 21 to 200 (large open-plan)	-	14 (1)

**Table 2:** Questionnaire layout.

Topic	ID	Question	Scale	Labels
Annoyance	Q1	How much does people chatting in your office annoy you?	5	Not at all (1)–Extremely (5)
Mental health and well-being (Feelings and symptoms)	Q2	What is the main feeling (or symptom) related to people chatting during your work tasks?	Single choice	○ Loss of concentration ○ Loss of motivation ○ Tiredness and overstrain ○ Stress ○ Anger ○ Negative feelings such as feeling displeased ○ Negative feelings toward other colleagues ○ Headache ○ None ○ Other
Work productivity	Q3	How much do you agree with the following statement? “People chatting around me often interrupts me during my work tasks”	5	Strongly disagree (1)–Strongly agree (5)
	Q4	How much do you agree with the following statement? “People chatting does not allow me to work as much as I would like to”		
	Q5	How much do you agree with the following statement? “People chatting around me significantly reduces my work performance”		
Mental health and well-being (Interpersonal relationships)	Q6	How much do you agree with the following statement? “People chatting compromises the harmony of the entire office”	5	Strongly disagree (1)–Strongly agree (5)
Occupants’ behavior (Personal strategies)	Q7	What is the main strategy that you use to reduce the annoyance resulting from people chatting?	Single choice	○ Change working space/room ○ Headphones with music ○ Noise cancelling headphones ○ Ask people to reduce voice ○ Change work task ○ Work from home ○ Take a break ○ Close the office door ○ None ○ Other
Presence of acoustic treatment	Q8	Are there any design strategies in your office aimed at the reduction of noise resulting from people chatting (sound absorption on ceiling or walls, partitions between desks, carpet, ecc.)?	Yes/No	
	Q9	If yes, what are the main strategies that are applied? (sound absorption on ceiling or walls, partitions between desks, carpets, ecc.)?	Multiple choice	○ Sound absorption on ceiling ○ Sound absorption on walls ○ Sound absorption on ceiling and walls ○ Partitions between desks ○ Carpets ○ None ○ Other
Occupants’ behavior (with reference to a warning system with lighting feedback)	Q10	Would you pay attention to a light-system that advises you and your colleagues to control your voice volume in order to reduce noise resulting from people chatting in your workplace?	Yes/No	

**Table 3:** Mean (Mn) and mode (Mo) values of the answers on noise annoyance, work productivity, and mental health and well-being related to ISN, for shared offices and open-plan offices, and two-tailed *p*-values of significance for the differences between the two office types, according to the MWU Test. Any statistically significant differences, with *p*-value < 0.05, are reported in bold.

Topic		Shared Offices (N = 597)		Open-Plan Offices (N = 481)		MWU *p*-Value
	ID	Mn	Mo	Mn	Mo	
**Noise annoyance**	**Q1**	2.54	2.00	3.07	3.00	**0.00**
**Work productivity**	**Q3**	3.06	3.00	3.44	4.00	**0.00**
	**Q4**	3.05	3.00	3.40	4.00	**0.00**
	**Q5**	2.98	3.00	3.22	4.00	**0.00**
**Mental health and well-being (Interpersonal relationships)**	**Q6**	2.71	2.00	2.98	3.00	**0.00**

**Table 4:** Mean (Mn) and mode (Mo) values of the answers on noise annoyance related to gender, age range, professional sector and city latitude, for shared (S) and open-plan (O) offices, and two-tailed *p*-values of significance of the differences according to the MWU or KW Test. Statistically significant differences with *p*-values < 0.05 are reported in bold. The following abbreviations are used for the professional sectors: TEC for “Technical”, EN-TE for “Engineering and Teaching”, MA-AD for “Management and Administration”, CR-DE-AR for “Creative, design and architecture”, SPA for “Sales and public affairs”, and OT for “Other”. The northern cities are Milan, Turin and Cuneo, and southern cities are Rome and Naples.

Sample		Descriptive Statistics	Shared Offices (N = 597)	Open-Plan Offices (N = 481)
**Gender**	**Female** **N(S) = 269,** **N(O) = 188**	**Mn**	2.51	3.19
		**Mo**	2.00	3.00
	**Male** **N(S) = 328,** **N(O) = 293**	**Mn**	2.58	2.99
		**Mo**	2.00	2.00
		**MWU *p*-value**	0.30	**0.04**
**Age range**	**18–35** **N(S) = 193,** **N(O) = 208**	**Mn**	2.36	2.92
		**Mo**	2.00	3.00
	**36–50** **N(S) = 187,** **N(O) = 98**	**Mn**	2.62	3.12
		**Mo**	2.00	3.00
	**51–65+** **N(S)** **= 217,** **N(O)** **= 175**	**Mn**	2.65	3.21
		**Mo**	3.00	3.00
		**KW *p-*value**	0.08	**0.03**
**Professional sector**	**TEC** **N(S)** **= 118, N(O) = 104**	**Mn**	2.62	3.18
		**Mo**	3.00	3.00
	**EN-TE** **N(S)** **= 181,** **N(O) = 126**	**Mn**	2.38	2.90
		**Mo**	2.00	2.00
	**MA-AD** **N(S)** **= 194,** **N(O) = 176**	**Mn**	2.60	3.10
		**Mo**	2.00	3.00
	**CR-DE-AR** **N(S)** **= 46,** **N(O) = 30**	**Mn**	2.57	2.93
		**Mo**	3.00	3.00
	**SPA** **N(S)** **= 9,** **N(O) = 20**	**Mn**	3.00	3.30
		**Mo**	2.00 and 4.00	3.00
	**OT** **N(S)** **= 49,** **N(O) = 25**	**Mn**	2.65	3.16
		**Mo**	2.00	3.00
		**KW *p-*value**	0.18	0.28
**City location**	**North** **N(S)** **= 480,** **N(O) = 416**	**Mn**	2.46	3.02
		**Mo**	2.00	3.00
	**South** **N(S)** **= 115,** **N(O) = 65**	**Mn**	2.90	3.77
		**Mo**	3.00	3.00
		**MWU *p*-value**	**0.00**	**0.01**

**Table 5:** Mean (Mn) and mode (Mo) values of the answers on noise annoyance related to the number of people in offices, and two-tailed *p*-value of significance of the difference between the number of people in offices, according to the KW Test. Statistically significant difference with *p*-value < 0.05 is reported in bold. The following abbreviations were used to indicate the type of office, on the basis of the number of people: S for “Shared office for two to five people”, MO for “Medium Open-plan office for six to 20 people”, LO for “Large Open-plan office for 21 to 200 people”.

Descriptive Statistics	Number of People in the Office			KW *p*-Value
	S (2–5) N = 597	MO (6–20) N = 467	LO (21–200) N = 14	
**Mn**	2.54	3.05	3.71	**0.00**
**Mo**	2.00	3.00	3.00	

**Table 6:** Mean (Mn) and mode (Mo) values of the answers on noise annoyance related to the self-estimated presence of acoustic treatment in shared and open-plan offices, and two-tailed *p*-values of significance of the difference between the presence and absence of treatment, according to the MWU Test, for both types of offices. Any statistically significant differences, with *p*-values < 0.05, are reported in bold.

**Sample**	**Descriptive Statistics**	**Presence of Acoustic Treatment**		**MWU** ***p*** **-Value**
		**Yes**	**No**	
		**N** **= 41**	**N** **= 556**	
**Shared Offices**	**Mn**	2.64	2.54	0.30
	**Mo**	3.00	2.00	
		**N = 96**	**N = 385**	
**Open-Plan Offices**	**Mn**	2.86	3.12	**0.02**
	**Mo**	2.00	3.00	

**Table 7:** Odds Ratio (OR) and 95% oconfidence interval (CI) of the covariates that significantly (*p*-values < 0.05) affect noise annoyance in shared and open-plan offices according to the logistic regression analysis.

Covariates (Reference Category)	Shared Offices (N = 597)			Open-Plan Offices (N = 481)		
	OR	95% CI	*p*-Value	OR	95% CI	*p*-Value
**Gender (Male)**	-	-	-	1.79	1.19–2.70	0.00
**City** **location (North)**	2.52	1.65–3.86	0.00	2.26	1.18–4.33	0.01
**Acoustic treatment (Absence for** **shared offices and Presence for** **open-plan offices)**	2.17	1.11–4.22	0.02	1.70	1.06–2.72	0.03
**Number of people in the office (6–20)**	-	-	-	8.70	1.11–68.20	0.04

## References

[1] Kaarlela-Tuomaala A., Helenius R., Keskinen E., Hongisto V. (2009). Effects of acoustic environment on work in private office rooms and open-plan offices—Longitudinal study during relocation. Ergonomics.

[2] Kang S., Ou D., Ming Mack C. (2017). The impact of indoor environmental quality on work productivity in university open-plan research offices. Build Environ..

[3] Hedge A. (1982). The open-plan office: A Systematic Investigation of Employee Reactions to Their Work Environment. Environ. Behav..

[4] Banbury S.P., Berry D.C. (2005). Office noise and employee concentration: Identifying causes of disruption and potential improvements. Ergonomics.

[5] Haapakangas A., Helenius R., Keskinen E., Hongisto V. Perceived acoustic environment, work performance and well-being—Survey results from Finnish offices. Proceedings of the 9th International Congress on Noise as a Public Health Problem.

[6] Pierrette M., Parizet E., Chevret P., Chatillon J. (2015). Noise effect on comfort in open-space offices: Development of an assessment questionnaire. Ergonomics.

[7] Hongisto V. (2005). A model predicting the effect of speech of varying intelligibility on work performance. Indoor Air.

[8] Jahncke H., Hygge S., Halin N., Green A.M., Dimberg K. (2011). Open-plan office noise: Cognitive performance and restoration. J. Environ. Psychol..

[9] Schlittmeier S.J., Liebl A. (2015). The effects of intelligible irrelevant background speech in offices—Cognitive disturbance, annoyance, and solutions. Facilities.

[10] Guski R., Felscher-Suhr U., Schuemer R. (1999). The concept of noise annoyance: How international experts see it. J. Sound Vib..

[11] Haapakangas A., Hongisto V., Hyönä J., Kokko J., Keränen J. (2014). Effects of unattended speech on performance and subjective distraction: The role of acoustic design in open-plan offices. Appl. Acoust..

[12] Varjo J., Hongisto V., Haapakangas A., Maula H., Koskela H., Hyönä J. (2015). Simultaneous effects of irrelevant speech, temperature and ventilation rate on performance and satisfaction in open-plan offices. J. Environ. Psychol..

[13] Martellotta F., della Crociata S., Simone A. Laboratory study on the effects of office noise on mental performance. Proceedings of the Forum Acusticum 2011.

[14] Public Health England (2013). North West Mental Wellbeing Survey 2012/13.

[15] Keyes C.L.M. (2002). The mental health continuum: From languishing to flourishing in life. J. Health Soc. Behav..

[16] World Health Organization (2000). Mental Health and Work: Impact, Issues and Good Practices.

[17] Pejtersen J., Allermann L., Kristensen T.S., Poulsen O.M. (2006). Indoor climate, psychosocial work environment and symptoms in open-plan offices. Indoor Air.

[18] Evans G.W., Johnson D. (2000). Stress and open-office noise. J. Appl. Psychol..

[19] De Croon E.M., Sluiter J.K., Kuijer P.P., Frings-Dresen M. (2005). The effect of office concepts on worker health and performance: A systematic review of the literature. Ergonomics.

[20] Danielsson C.B. (2005). Office Environment, Health & Job Satisfaction. An Explorative Study of Design’s Influence.

[21] Brennan A., Chugh J.S., Kline T. (2002). Traditional versus open office design—A longitudinal field study. Environ. Behav..

[22] Frontczak M., Schiavon S., Goins J., Arens E., Zhang H., Wargocki P. (2012). Quantitative relationships between occupant satisfaction and satisfaction aspects of indoor environmental quality and building design. Indoor Air.

[23] Sakellaris I.A., Saraga D.E., Mandin C., Roda C., Fossati S., De Kluizenaar Y., Carrer P., Dimitroulopoulou S., Mihucz V.G., Szigeti T. (2016). Perceived indoor environment and occupants’ comfort in European “Modern” office buildings: The OFFICAIR Study. Int. J. Environ. Res. Public Health.

[24] Seddigh A., Berntson E., Jönsson F., Danielson C.B., Westerlund H. (2015). Effect of variation in noise absorption in open-plan office: A field study with a cross-over design. J. Environ. Psychol..

[25] Hongisto V., Haapakangas A., Varjo J., Helenius R., Koskela H. (2016). Refurbishment of an open-plan office—Environmental and job satisfaction. J. Environ. Psychol..

[26] Haapakangas A., Kankkunen E., Hongisto V., Virjonen P., Oliva D., Keskinen E. (2011). Effects of five speech masking sounds on performance and acoustic satisfaction. Implications for open-plan offices. Acta Acust. (United Acust.).

[27] Renz T., Leistner P., Liebl A. (2018). Auditory distraction by speech: Comparison of fluctuating and steady speech-like masking sounds. J. Acoust. Soc. Am..

[28] Di Blasio S., Vannelli G., Shtrepi L., Masoero M.C., Astolfi A. A subjective investigation on the impact of irrelevant speech noise on health, well-being and productivity in open-plan offices. Proceedings of the Euronoise 2018.

[29] SoundEar. https://soundear.com/.

[30] Bradley J.S. (2003). The acoustical design of conventional open plan offices. Can. Acoust..

[31] Carboni O.A., Russu P. (2018). Measuring and forecasting regional environmental and economic efficiency in Italy. Appl. Econ..

[32] Nulty D.D. (2008). The adequacy of response rates to online and paper surveys: What can be done?. Assess. Eval. High. Ed..

[33] Google Forms. https://www.google.com/forms/about/.

[34] World Health Organization (2005). Mental Health Policies and Programmes in the Workplace.

[35] De Dear R., Brager G. (1998). Developing an adaptive model of thermal comfort and preference. ASHRAE Trans..

[36] Nicol J.F., Humphreys M.A. (2002). Adaptive thermal comfort and sustainable thermal standards for buildings. Energy Build..

[37] Ortalda F. (1998). La Survey in Psicologia (“The Survey in Psychology”).

[38] Converse J.M., Presser S. (1986). Survey Questions: Handcrafting the Standardized Questionnaire.

[39] Sigel S., Castellan N.J. (1988). Non Parametric Statistics for the Behavioral Sciences.

[40] Bursac Z., Gauss C.H., Williams D.K., Hosmer D.W. (2008). Purposeful selection of variables in logistic regression. Source Code Biol. Med..

[41] Fleiss J.L., Levin B., Cho Paik M. (2003). Statistical Methods for Rates and Proportions.

[42] Danielsson C.B., Bodin L., Wulff C., Theorell T. (2015). The relation between office type and workplace conflict: A gender and noise perspective. J. Environ. Psychol..

[43] Logie R.H., Baddeley A.D. (1987). Cognitive processes in counting. J. Exp. Psychol. Learn. Mem. Cogn..

[44] (2012). ISO 3382-3:2012. Acoustics—Measurement of Room Acoustic Parameters—Part 3: Open-Plan Offices.

[45] Brisson C., Blanchette C., Guimont C., Dion G., Moisan J., Vézina M., Dagenais G.R., Mǎsse L. (1998). Reliability and validity of the French version of the 18-item Karasek Job Content Questionnaire. Work Stress.

[46] Schutte M., Marks A., Wenning E., Griefahn B. (2007). The development of the noise sensitivity questionnaire. Noise Health.

[47] Wright K.B. (2005). Researching Internet-Based Populations: Advantages and Disadvantages of Online Survey Research, Online Questionnaire Authoring Software Packages, and Web Survey Services. J. Comput. Mediat. Commun..

[48] Sax L.J., Gilmartin S.K., Bryant A.N. (2003). Assessing response rate and nonresponse bias in web and paper surveys. Res. High. Ed..

